# Short-term effectiveness of HIV care coordination among persons with recent HIV diagnosis or history of poor HIV outcomes

**DOI:** 10.1371/journal.pone.0204017

**Published:** 2018-09-24

**Authors:** Denis Nash, McKaylee M. Robertson, Kate Penrose, Stephanie Chamberlin, Rebekkah S. Robbins, Sarah L. Braunstein, Julie E. Myers, Bisrat Abraham, Sarah Kulkarni, Levi Waldron, Bruce Levin, Mary K. Irvine

**Affiliations:** 1 Institute for Implementation Science in Population Health, City University of New York, New York, NY United States of America; 2 Graduate School of Public Health and Health Policy, City University of New York, New York, NY United States of America; 3 Bureau of HIV/AIDS, New York City Department of Health and Mental Hygiene, New York, NY United States of America; 4 Division of Infectious Diseases, Department of Medicine, Columbia University College of Physicians and Surgeons, New York, NY United States of America; 5 Department of Biostatistics, Mailman School of Public Health, Columbia University, New York, NY United States of America; Hospital for Sick Children, CANADA

## Abstract

The New York City HIV Care Coordination Program (CCP) combines multiple evidence-based strategies to support persons living with HIV (PLWH) at risk for, or with a recent history of, poor HIV outcomes. We assessed the comparative effectiveness of the CCP by merging programmatic data on CCP clients with population-based surveillance data on all New York City PLWH. A non-CCP comparison group of similar PLWH who met CCP eligibility criteria was identified using surveillance data. The CCP and non-CCP groups were matched on propensity for CCP enrollment within four baseline treatment status groups (newly diagnosed or previously diagnosed and either consistently unsuppressed, inconsistently suppressed or consistently suppressed). We compared CCP to non-CCP proportions with viral load suppression at 12-month follow-up. Among the 13,624 persons included, 15∙3% were newly diagnosed; among the 84∙7% previously diagnosed, 14∙2% were consistently suppressed, 28∙9% were inconsistently suppressed, and 41∙6% were consistently unsuppressed in the year prior to baseline. At 12-month follow-up, 59∙9% of CCP and 53∙9% of non-CCP participants had viral load suppression (Relative Risk = 1.11, 95%CI:1.08–1.14). Among those newly diagnosed and those consistently unsuppressed at baseline, the relative risk of viral load suppression in the CCP versus non-CCP participants was 1.15 (95%CI:1.09–1.23) and 1.32 (95%CI:1.23–1.42), respectively. CCP exposure shows benefits over no CCP exposure for persons newly diagnosed or consistently unsuppressed, but not for persons suppressed in the year prior to baseline. We recommend more targeted case finding for CCP enrollment and increased attention to viral load suppression maintenance.

## Introduction

The goal of HIV treatment is to achieve viral load suppression (VLS), which occurs when the amount of viral load (VL) circulating in the body is very low (no higher than 200 HIV RNA copies/μL).[1] The HIV care continuum represents the series of sequential steps of HIV medical care engagement that persons living with HIV (PLWH) go through in order to achieve VLS and is typically depicted as the number or proportion of persons who are estimated to be (1) HIV-infected, (2) HIV-diagnosed, (3) receiving HIV-medical care, (4) prescribed antiretroviral treatment (ART) and (5) virally suppressed.[1–3]

HIV care continuum outcomes remain suboptimal throughout the US [4]. In 2013, an estimated 1∙1 million persons were living with HIV infection; an estimated 87% were diagnosed; and 55% of persons diagnosed achieved viral load suppression (VLS) [4]. Efforts aimed at controlling the domestic HIV epidemic will require integrated medical and social support approaches in order to extend the benefits of HIV treatment to the large numbers of PLWH who to date have not been able to achieve and sustain VLS [5].

A first step toward strengthening the HIV care continuum is to address immediate barriers to adherence and to improve short-term outcomes among PLWH who are under-engaged with HIV medical care and treatment. HIV affects vulnerable and marginalized populations, and PLWH have higher rates of mental illness, substance use disorders, and unstable housing, all serious and often co-occurring barriers to achieving desired HIV outcomes [6, 7]. Research has shown that services specifically addressing these barriers can facilitate improved outcomes along the care continuum [8–13]. However, few studies have utilized a contemporaneous comparison group to examine whether HIV case management programs are more effective than usual care approach (or other interventions) at promoting VLS [8, 14–21]. Two cohort studies have evaluated VLS outcomes and found no program benefit for VLS [15, 18].

We conducted an observational study to assess the comparative effectiveness of a comprehensive HIV care coordination intervention on VLS outcomes among persons in New York City (NYC) with documented barriers to HIV care and treatment engagement [8].

## Materials and methods

### Intervention description

In December 2009, with Ryan White Part A funding, the NYC Department of Health and Mental Hygiene (DOHMH) launched the HIV Care Coordination Program (CCP) to support persons at high risk for, or with a recent history of, poor HIV care outcomes.

CCP eligibility criteria permit enrollment of HIV-infected adults or emancipated minors who are eligible for local Ryan White Part A services (based on residence within the New York grant area and household income <435% of federal poverty level) and who are (1) newly diagnosed with HIV; (2) never in care or lost to care for at least 9 months; (3) irregularly in care or often missing appointments; (4) starting a new ART regimen; (5) experiencing ART adherence barriers; or (6) manifesting treatment failure or ART resistance.[14] The CCP combines various evidence-based programmatic elements including case management, patient navigation, directly observed therapy (DOT), structured health promotion in home/field visits, and outreach to assist patients in accessing medical care and support services, such as mental health treatment, substance use treatment, and housing assistance. The intensity and focus of these services can be tailored to meet individual needs and circumstances. The intervention has previously been described, and CCP materials are available on the DOHMH website [14, 22].

Importantly, the CCP was rolled out purely as a service program, with no randomization or contemporaneous control/comparison group. Early assessments of CCP outcomes used individuals as their own historical controls (i.e., pre-post) [8, 14, 22]. While these assessments offered preliminary evidence suggestive of program effectiveness, they could not distinguish program effects from secular improvements in VLS in NYC during the same time period.[23] Focusing on the last viral load (VL) in a 12-month follow-up period, this study aimed to compare VLS proportions between CCP clients and demographically and clinically similar PLWH who, during the same time period, were eligible for but did not enroll in the CCP (“non-CCP PLWH”).

### Data sources

We retrospectively constructed an observational cohort of persons enrolled and not enrolled in the CCP by merging provider-reported programmatic data with data from the longitudinal population-based NYC HIV Surveillance Registry (“the Registry”). Our approach to constructing an observational cohort using the Registry has previously been detailed.[24] The Registry contains demographic and laboratory information on *all* diagnoses of HIV (since 2000) and AIDS (since 1981) reported in NYC, with the addition of comprehensive and *longitudinal* HIV-related laboratory reporting (including *all* CD4-lymphocyte [CD4] and VL test results) starting in 2005. The Registry does not contain direct measures of primary care or HIV treatment status [25]. However, validation work by DOHMH [26, 27] has shown that VL/CD4 tests reported to surveillance are reliable indicators of receipt of HIV care in NYC, and laboratory reporting is considered to be ~99% complete. Vital status information is updated through regular matches with local and national death data. Data on CCP client enrollments were drawn from the DOHMH Electronic System for HIV/AIDS Reporting and Evaluation (eSHARE), a secure, Web-based, named programmatic reporting system.

In eSHARE, we identified all persons who enrolled in the CCP from December 1, 2009 to March 31, 2013. The 2013 cut-off was chosen so that we would have adequate power to detect a modest effect as statistically significant and so that we would be able to examine and compare viral suppression over the short-term (as reported here) and long-term (analyses extending out to March 2017), with the same cohort. Using data reported to the Registry as of September 30, 2014, we identified all persons who were diagnosed with HIV as of March 31, 2013, were living 12 months after diagnosis, were at least 18 years old as of March 31, 2013, and had at least one CD4 or VL result dated between December 1, 2007 and March 31, 2013. To ensure adequate outcome observation time, we excluded CCP clients who died within 12 months of program enrollment (n = 279).

This study was approved by the institutional review boards at The City University of New York and the New York City Department of Health and Mental Hygiene. For these secondary analyses of de-identified data, we received a waiver for informed consent under 45 CFR 46.116(d)(2).

### 'Non-CCP PLWH' comparison group

We constructed a non-CCP comparison group of PLWH who were similar to CCP enrollees in four steps. First, through the NYC Registry match, we identified PLWH who were not enrolled in the CCP but met broad clinical eligibility criteria for CCP enrollment at one or more times (CCP eligibility window) during December 1, 2009 to March 31, 2013. Second, we assigned eligible non-CCP PLWH pseudo-enrollment dates falling within their windows of CCP eligibility. Third, we limited to PLWH with evidence of recent NYC HIV medical care (≥1 CD4 or VL test reported to the Registry in the 24 months after the pseudo-enrollment date). Finally, we matched CCP enrollees to non-CCP PLWH according to baseline treatment status, enrollment/pseudo-enrollment dates and propensity for enrollment in the CCP.

#### Step 1) identify persons meeting broad CCP eligibility criteria

Using information from the Registry, we identified persons as eligible for enrollment in the CCP if they were 1) *newly diagnosed* with HIV from December 1, 2008 to March 31, 2013; 2) *out of medical care*, defined as lacking CD4 and VL laboratory monitoring for any nine-month post-diagnosis period during December 1, 2007 to March 31, 2013; 3) *treatment naïve*, defined as ever having a CD4 count <200 reported as of March 31, 2013, but not initiating antiretroviral treatment [ART] (never having a ≥1-log drop in VL within 3 months, or an unsuppressed VL [>200 copies/μL] followed by a suppressed VL [≤200 copies/μL]) as of the date of a CD4 count <200 [25]; 4) *exhibiting poor ART adherence* as of March 31, 2012, defined as not achieving VLS or not having any VL tests reported in the first 12 months after ART initiation (a ≥1-log drop in VL within 3 months, or an unsuppressed VL followed by a suppressed VL) [25]; 5) *experiencing viral rebound* (a suppressed VL followed by 2 consecutive unsuppressed VL tests in the 12 months following the suppressed VL, from December 1, 2007 to March 31, 2013); or 6) *registering a high VL* (≥10,000 copies/μL) from December 1, 2008 to March 31, 2013.

#### Step 2) assign eligible persons in the non-CCP PLWH comparison group a pseudo-enrollment date

Non-CCP PLWH who met any of the eligibility criteria were assigned an *eligibility window* (S1 Table), or a range of time between December 2009 and March 2013 (the CCP enrollment period), during which they met the above CCP eligibility criteria. For example, persons were considered eligible as “newly diagnosed” during the 12 months following diagnosis. Persons could be assigned multiple eligibility windows based on qualifying for the CCP via multiple Registry criteria and/or qualifying under the same criterion multiple times. To identify a start of follow-up for each member of the comparison group (i.e., time zero from which to prospectively assess outcomes in comparison to those in the CCP group), we randomly assigned each non-CCP PLWH a pseudo-enrollment date that fell within one of their eligibility windows. Further, to control for secular trends in VLS, pseudo-enrollment dates were assigned with probabilities such that their temporal distribution matched that of the enrollment dates among CCP enrollees (i.e., frequency matching). For persons who died, eligibility windows ended at least 12 months prior to the date of death, to ensure 12 months for outcome observation following the pseudo-enrollment date.

#### Step 3) identify NYC medical care recipients in the non-CCP PLWH group

After pseudo-enrollment dates were assigned, we restricted the eligible pool to persons who had at least one valid CD4 or VL test reported to the Registry in the 24 months after the pseudo-enrollment date. We required one laboratory test to identify persons accessing HIV medical care in NYC after the pseudo-enrollment date, as CCP enrollment and service initiation entails connection to NYC HIV medical care [28].

#### Step 4) propensity model and match

After constructing this eligible non-CCP-enrolled population, we prepared to match persons in the non-CCP PLWH comparison group to those in the CCP group using baseline treatment status, propensity scores, and enrollment/pseudo-enrollment dates. Given that 12-month VLS outcomes would be expected to differ by baseline treatment status/engagement, we created four mutually exclusive baseline treatment status groups: 1) newly diagnosed (in the 12 months prior to enrollment/pseudo-enrollment date), 2) consistently suppressed (≥2 VLs ≥90 days apart, and all VLs ≤200 copies/μL, in the 12 months prior to enrollment/pseudo-enrollment date), 3) consistently *un*suppressed (all VLs reported >200 copies/μL or no VLs reported in the 12 months prior to enrollment/pseudo-enrollment), or 4) inconsistently suppressed (at least 1 VL ≤200 copies/μL, but *not all* VLs ≤200 copies/μL, in the 12 months prior to enrollment/pseudo-enrollment).

We used logistic regression to estimate the propensity for enrollment in the CCP within each of the above 4 groups. We combined two baseline treatment status groups, groups 3 and 4, for one propensity score model, because we hypothesized the propensity of enrollment in the CCP would be influenced by the same potential confounders for these two groups; when we estimated the propensity for enrollment with individual models for groups 3 and 4, the effect estimates from the pooled model did not differ from the effect estimates from the individual models. We report the results for the pooled model because we were able to match a greater proportion of CCP enrollees, enhancing generalizability. Subsequent matching occurred within each of the four groups.

For the three propensity score models, we started with a model that included all of our *a priori* hypothesized and measured confounders and used backward selection to identify the model with the lowest value of Akaike’s Information Criterion (AIC). The variables that we suspected were confounders of the relationship between CCP enrollment and the VLS outcome were sex, race/ethnicity, age at enrollment/pseudo-enrollment, country of birth, HIV transmission risk, year of diagnosis, baseline VL, baseline CD4, successful linkage to HIV care within three months of diagnosis, presence of an AIDS diagnosis within one year of HIV diagnosis, number of VL laboratory tests reported in the year prior to enrollment/pseudo-enrollment, residential ZIP code at enrollment/pseudo-enrollment, HIV prevalence and poverty level within ZIP code at enrollment/pseudo-enrollment, and interaction terms for baseline CD4 and baseline VL, baseline CD4 and race, sex and risk, and year of diagnosis and risk. Per the American Community Survey, poverty level within the residential ZIP code at enrollment/pseudo-enrollment was classified as high (poverty greater than the median poverty level for a given year of enrollment/pseudo-enrollment) versus low. HIV prevalence was based on aggregated NYC HIV surveillance data for the ZIP code by year of enrollment/pseudo-enrollment, and was classified as high (prevalence greater than the median HIV prevalence for a given year) versus low.

Within each of the four baseline treatment status groups, we matched on propensity scores and enrollment/pseudo-enrollment dates (± 3 months). We used a 1:1 'greedy' match technique, and the match algorithm proceeded sequentially from 8 to 1 decimal places of the propensity score [29, 30]. We considered a standardized difference of ≥0∙1 to indicate an imbalance in the measured confounders between the CCP and non-CCP groups [31]. The final match included no imbalances ≥0∙1.

### Outcome definition and Care Coordination Program effectiveness estimate

The VLS outcome was based on the last VL laboratory result reported to the Registry in the 12 months following the enrollment/pseudo-enrollment date, and was dichotomized as ≤200 or >200 copies/μL. Persons with no VL in the Registry for the entire 12-month follow-up period were classified as not having VLS. While rare, 1∙3% (87/6,812) of the CCP and 5∙4% (353/6,812) of the non-CCP PLWH were missing a VL result. We used a GEE model with binary error distribution and identity link to estimate the difference in proportion of CCP and non-CCP participants with VLS, accounting for the matched-pairs design. The arithmetic difference was expressed in percentage point units. We used a GEE model with binary error distribution and log link to estimate the relative effect of the CCP on VLS, accounting for the matching. Absolute differences and relative risks were estimated with the GENMOD procedure in SAS version 9∙3 (SAS Institute, Cary, N.C.) The absolute and relative effect of the CCP was estimated for each of the four baseline treatment status groups.

## Results

**Fig 1:**
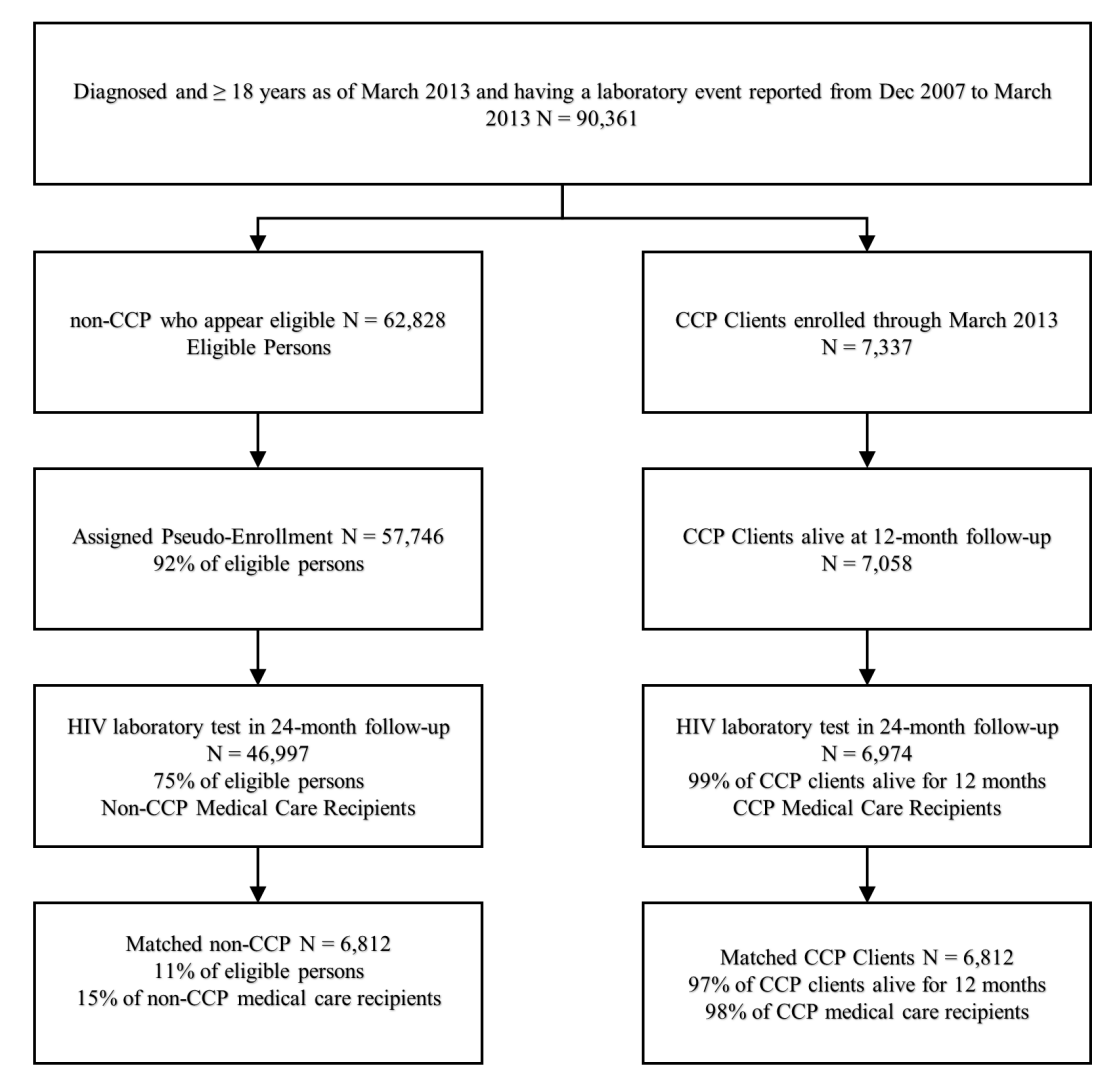
Inclusion in the Care Coordination and non-care coordination comparison group analysis, New York city, 2009–2013.

From December 1, 2009 to March 31, 2013, a total of 7,337 persons enrolled in the CCP; of those, 7,058 (96∙2%) were still living 12 months after enrollment. Of the 62,828 non-enrolled PLWH who were eligible for enrollment in the CCP, 91∙9% (57,746) were assigned a pseudo-enrollment date; 74∙8% (46,997) had an HIV-related laboratory test in NYC in the two years following their pseudo-enrollment date; and 10∙8% (6,812) were matched to a CCP enrollee, allowing us to include 96∙5% (6,812/7,058) of CCP clients eligible for this analysis (Fig 1).

Prior to propensity score matching, the CCP and non-CCP groups differed substantially by measured demographic and clinical characteristics (Table 1). After propensity score matching, the CCP and non-CCP groups were similar on all measured characteristics (Table 1). In terms of baseline treatment status, 15∙3% were newly diagnosed in the year prior to enrollment/pseudo-enrollment, and among the 84∙7% who were previously diagnosed, 14∙2% were consistently virally suppressed, 28∙9% were inconsistently suppressed, and 41∙6% were consistently *un*suppressed in the year prior to enrollment. Only 31∙3% were virally suppressed at the time of last reported lab measurement prior to enrollment/pseudo-enrollment, and the median CD4 count was 314 cells/μL (IQR 150–506), with 31∙8% having CD4<200 cells/μL.

**Table 1:** Pre and post-match demographic and clinical characteristics of Care Coordination and non-care coordination program persons—New York city, 2009–2013.

	Pre-Match			Post-Match		
	Total	non-CCP	CCP	Total	non-CCP	CCP
Total	53,971 (100.0)	46,997 (100.0)	6,974 (100.0)	13,624 (100.0)	6,812 (100.0)	6,812 (100.0)
Sex						
Male	38,380 (71.1)	33,920 (72.2)	4,460 (64.0)	8,750 (64.2)	4,379 (64.3)	4,371 (64.2)
Female	15,591 (28.9)	13,077 (27.8)	2,514 (36.0)	4,874 (35.8)	2,433 (35.7)	2,441 (35.8)
Race/Ethnicity						
Black	25,646 (47.5)	21,931 (46.7)	3,715 (53.3)	7,248 (53.2)	3,603 (52.9)	3,645 (53.5)
Hispanic	17,339 (32.1)	14,682 (31.2)	2,657 (38.1)	5,260 (38.6)	2,678 (39.3)	2,582 (37.9)
White	9,766 (18.1)	9,338 (19.9)	428 (6.1)	820 (6.0)	395 (5.8)	425 (6.2)
Other/Unknown	1,220 (2.2)	1,046 (2.3)	174 (2.5)	296 (2.2)	136 (2.0)	160 (2.3)
Age Category (years)						
≤24	3,551 (6.6)	2,999 (6.4)	552 (7.9)	1,070 (7.9)	534 (7.8)	536 (7.9)
25–44	21,931 (40.6)	18,985 (40.4)	2,946 (42.2)	5,820 (42.7)	2,944 (43.2)	2,876 (42.2)
45–64	26,540 (49.2)	23,288 (49.6)	3,252 (46.6)	6,301 (46.2)	3,113 (45.7)	3,188 (46.8)
65+	1,949 (3.6)	1,725 (3.7)	224 (3.2)	433 (3.2)	221 (3.2)	212 (3.1)
Transmission Risk						
Men who have sex with men	20,440 (37.9)	18,410 (39.2)	2,030 (29.1)	3,986 (29.3)	1,995 (29.3)	1,991 (29.2)
Injection drug use history	8,916 (16.5)	7,433 (15.8)	1,483 (21.3)	2,916 (21.4)	1,474 (21.6)	1,442 (21.2)
Heterosexual	11,832 (21.9)	9,930 (21.1)	1,902 (27.3)	3,678 (27.0)	1,836 (27.0)	1,842 (27.0)
Other/unknown	12,783 (23.7)	11,224 (23.9)	1,559 (22.4)	3,044 (22.3)	1,507 (22.1)	1,537 (22.6)
Country of Birth						
US/US dependency	35,563 (65.9)	30,932 (65.8)	4,631 (66.4)	9,127 (67.0)	4,571 (67.1)	4,556 (66.9)
Foreign born	9,764 (18.1)	8,160 (17.4)	1,604 (23.0)	3,022 (22.2)	1,498 (22.0)	1,524 (22.4)
Unknown	8,644 (16.0)	7,905 (16.8)	739 (10.6)	1,475 (10.8)	743 (10.9)	732 (10.7)
Year of HIV Diagnosis						
Prior 1995	9,989 (18.5)	8,689 (18.5)	1,300 (18.6)	2,529 (18.6)	1,258 (18.5)	1,271 (18.7)
1995–1999	9,976 (18.5)	8,738 (18.6)	1,238 (17.8)	2,396 (17.6)	1,181 (17.3)	1,215 (17.8)
2000–2004	14,684 (27.2)	12,862 (27.4)	1,822 (26.1)	3,621 (26.6)	1,813 (26.6)	1,808 (26.5)
2005–2009	11,549 (21.4)	10,120 (21.5)	1,429 (20.5)	2,858 (21.0)	1,464 (21.5)	1,394 (20.5)
2010–2013	7,773 (14.4)	6,588 (14.0)	1,185 (17.0)	2,220 (16.3)	1,096 (16.1)	1,124 (16.5)
Baseline Viral Load (copies/μL)						
>1500	17,634 (32.7)	13,820 (29.4)	3,814 (54.7)	7,374 (54.1)	3,666 (53.8)	3,708 (54.4)
>200–1499	6,749 (12.5)	6,039 (12.8)	710 (10.2)	1,423 (10.4)	713 (10.5)	710 (10.4)
≤200	21,108 (39.1)	18,927 (40.3)	2,181 (31.3)	4,266 (31.3)	2,141 (31.4)	2,125 (31.2)
No viral load	8,480 (15.7)	8,211 (17.5)	269 (3.9)	561 (4.1)	292 (4.3)	269 (3.9)
Baseline CD4 Count (cells/μL)						
<200	8,758 (16.2)	6,466 (13.8)	2,292 (32.9)	4,335 (31.8)	2,135 (31.3)	2,200 (32.3)
200–349	9,182 (17.0)	7,684 (16.3)	1,498 (21.5)	2,953 (21.7)	1,486 (21.8)	1,467 (21.5)
350–499	9,668 (17.9)	8,469 (18.0)	1,199 (17.2)	2,416 (17.7)	1,228 (18.0)	1,188 (17.4)
500+	18,392 (34.1)	16,678 (35.5)	1,714 (24.6)	3,356 (24.6)	1,668 (24.5)	1,688 (24.8)
No CD4	7,971 (14.8)	7,700 (16.4)	271 (3.9)	564 (4.1)	295 (4.3)	269 (3.9)
Initiated Care ≤91 Days						
No	37,412 (69.3)	32,701 (69.6)	4,711 (67.6)	9,243 (67.8)	4,615 (67.7)	4,628 (67.9)
Yes	16,559 (30.7)	14,296 (30.4)	2,263 (32.4)	4,381 (32.2)	2,197 (32.3)	2,184 (32.1)
Baseline HIV Prevalence & Poverty						
High poverty & high prevalence	28,689 (53.2)	23,985 (51.0)	4,704 (67.5)	9,240 (67.8)	4,647 (68.2)	4,593 (67.4)
Low poverty & high prevalence	12,473 (23.1)	11,227 (23.9)	1,246 (17.9)	2,412 (17.7)	1,188 (17.4)	1,224 (18.0)
High poverty & low prevalence	1,740 (3.2)	1,495 (3.2)	245 (3.5)	459 (3.4)	227 (3.3)	232 (3.4)
Low poverty & low prevalence	7,003 (13.0)	6,327 (13.5)	676 (9.7)	1,311 (9.6)	650 (9.5)	661 (9.7)
Unknown	4,066 (7.5)	3,963 (8.4)	103 (1.5)	202 (1.5)	100 (1.5)	102 (1.5)
Number of Viral Load Labs in year prior to enrollment						
0 VL labs	8,480 (15.7)	8,211 (17.5)	269 (3.9)	561 (4.1)	292 (4.3)	269 (3.9)
1–3 VL labs	28,923 (53.6)	25,169 (53.6)	3,754 (53.8)	7,404 (54.3)	3,729 (54.7)	3,675 (53.9)
4+ VL labs	16,568 (30.7)	13,617 (29.0)	2,951 (42.3)	5,659 (41.5)	2,791 (41.0)	2,868 (42.1)
Baseline Treatment Status						
Newly diagnosed ^a^	7,682 (14.2)	6,590 (14.0)	1,092 (15.7)	2,088 (15.3)	1,044 (15.3)	1,044 (15.3)
Consistently suppressed ^b^	5,939 (11.0)	4,917 (10.5)	1,022 (14.7)	1,934 (14.2)	967 (14.2)	967 (14.2)
Consistently *un*suppressed ^c^	21,631 (40.1)	18,742 (39.9)	2,889 (41.4)	5,666 (41.6)	2,833 (41.6)	2,833 (41.6)
Inconsistently suppressed ^d^	18,719 (34.7)	16,748 (35.6)	1,971 (28.3)	3,936 (28.9)	1,968 (28.9)	1,968 (28.9)

^a.^ Newly diagnosed within 12 months of enrollment or pseudo-enrollment

^b.^ Consistently suppressed: at least 2 VLs at least 90 days apart and all VLs < 200 copies/μL in 12 months prior to enrollment or pseudo-enrollment

^c.^ Consistently *un*suppressed: All labs reported >200 in 12 months prior to enrollment or pseudo-enrollment. This includes persons missing VL labs. Persons with 1 lab >200 would be in this group.

^d.^ Inconsistently suppressed: Previously diagnosed and not in groups (b) and (c) above. Persons with 1 lab ≤200 would be in this group.

By twelve months after enrollment/pseudo-enrollment, 59∙9% of the CCP group and 53∙9% of the non-CCP comparison group were suppressed on their most recent VL test (Table 2). The proportion of persons with VLS differed by baseline treatment status. Two baseline treatment status groups showed significantly higher CCP versus non-CCP VLS in the follow-up year: newly diagnosed PLWH (73∙3% versus 63∙3%, respectively; Absolute Difference as a percentage (AD): 9∙96 (95%CI 6∙09–13∙83); Relative Risk (RR):1∙15 (95%CI 1∙09–1∙23); and PLWH who were previously diagnosed and consistently *un*suppressed in the year prior to enrollment/pseudo-enrollment (42∙5% vs. 32∙1%, respectively; AD:10∙41 (95%CI 7∙94–12∙89); RR:1∙32 (95%CI 1∙23–1∙42)). No differences in VLS were observed among persons with consistent VLS or with inconsistent VLS in the year prior to enrollment/pseudo-enrollment (91∙7% vs. 90∙6%; AD_consistent VLS_ = 1∙14 (95%CI -1∙42–3∙69); RR_consistent VLS_ = 1∙01 (95%CI 0∙98–1∙04); and 62∙2% vs. 62∙3%; AD_inconsistent VLS_ = -0∙10 (95%CI: -3∙08–2∙88); RR_inconsistent VLS_ = 0∙99 (95%CI: 0∙95–1∙05), respectively).

**Table 2:** Relative and absolute difference for having viral load suppression at 12-month post measure (Care Coordination Program versus non-care coordination program persons)–New York city, 2009–2013.

	Total	% VLS Post^a^	N Denominator (for CCP or non-CCP)	CCP % VLS Post^a^	Non-CCP % VLS Post^a^	Arithmetic Difference in Percentage Points (95% Confidence Intervals (CI))	Relative Risk (95% CI)	P-Value^b^
**Overall**	**13,624**	**56.9**	**6,812**	**59.9**	**53.9**	5.99 (4.49, 7.49)	**1.11 (1.08, 1.14)**	<0.001
**Baseline Treatment Status**								
Newly diagnosed ^c^	2,088	68.3	1,044	73.3	63.3	9.96 (6.09, 13.83)	**1.15 (1.09, 1.23)**	<0.001
Consistently suppressed ^d^	1,934	91.2	967	91.7	90.6	1.14 (-1.42, 3.69)	1.01 (0.98, 1.04)	0.38
Consistently unsuppressed ^e^	5,666	37.3	2,833	42.5	32.1	10.41 (7.94, 12.89)	**1.32 (1.23, 1.42)**	<0.001
Inconsistently suppressed ^f^	3,936	62.3	1,968	62.2	62.3	-0.10 (-3.08, 2.88)	0.99 (0.95, 1.05)	0.95

CCP: Care Coordination Program, CI: Confidence Intervals, VLS: Viral Load Suppression

^a.^ Proportion with the last viral load (≤200) in the 12 months after enrollment or pseudo-enrollment. Persons missing a VL are considered to have unsuppressed VL (>200)

^b.^P-values for the arithmetic difference in proportions and their relative risks did not differ to the number of decimal places shown

^c.^ Newly diagnosed within 12 months of enrollment or pseudo-enrollment

^d.^ Consistently suppressed: at least 2 VLs at least 90 days apart and all VLs < 200 copies/μL in 12 months prior to enrollment or pseudo-enrollment

^e.^ Consistently *un*suppressed: All labs reported >200 in 12 months prior to enrollment or pseudo-enrollment. This includes persons missing VL labs. Persons with 1 lab >200 would be in this group.

^f.^ Inconsistently suppressed: Previously diagnosed and not in groups (d) and (e) above. Persons with 1 lab ≤200 would be in this group.

## Discussion

Using a surveillance-based method for comparison group selection in an observational effectiveness study, we found New York’s Ryan White CCP intervention to have a significant positive effect on VLS for newly diagnosed persons and for previously diagnosed persons who were consistently *un*suppressed in the year prior to enrollment. Previously, we have shown a positive CCP effect using a single-group pre-post assessment, in which the proportion of CCP enrollees with VLS increased from 32.3% in the year prior to enrollment to 50.9% in the year after enrollment.[8, 14] However, the single-group pre-post design could not isolate program effects from secular improvements in VLS (i.e., annual citywide improvements in VLS that occurred in tandem with population-based HIV treatment strategies).[8, 14, 32, 33] A strength of our contemporaneous comparison group approach is that it accounts for these secular trends in VLS; these data suggest the CCP may help PLWH with initial hurdles to ART access and adherence, and point to the potential value of targeting the intervention to those individuals who have not previously achieved VLS. However, twelve months after CCP enrollment, there remained substantial room for improvement in VLS among those CCP participants who were *un*suppressed throughout the year prior to enrollment, as only 42.5% had achieved VLS on their last VL measurement.

There was a high prevalence of advanced HIV disease in the cohort (31∙8% had CD4<200 cells/uL), with a substantial proportion of persons not suppressed on their last VL measure in the 12-month follow-up period (43∙1%). This likely reflects the persistence of major barriers to care and treatment engagement in our study population. For the sample of 7,058 CCP clients alive at 12-month follow-up, we previously described the baseline prevalence of psychosocial barriers, including unstable housing (22∙1%), low mental health functioning (30∙0%), and self-reported recent hard drug use (15∙1%); half of CCP clients (50∙4%, or 3,556) had documentation of at least one of these barriers at the time of enrollment, and only 24∙4% of the 3,556 (or 38∙6% of the 2,250 with a follow-up assessment) were documented as having none of those barriers at last assessment in the 12 months post-enrollment [8]. Thus, housing, mental health and/or drug-related barriers persisted for ≥61∙4% (and up to 75∙6%) of clients presenting with those barriers at CCP enrollment.

This proportion remains high because fully resolving psychosocial barriers takes time and repeated efforts.[34, 35] Further, estimates of barrier resolution are conservative.[8] For example, clients may be receiving harm reduction assistance through the CCP but not have stopped using drugs entirely. For clients enrolling with more than one barrier, the CCP may have resolved one barrier but not all barriers by 12 months, and clients with fewer total barriers would still be considered to have persistent barriers. In the single-group pre-post evaluation, resolution of barriers was associated with VLS improvement; specifically, clients who obtained stable housing post-baseline showed significantly greater VLS improvement than those remaining in unstable housing.[8] Thus, short of eliminating barriers altogether, the CCP may mitigate or reduce their effects through case management, adherence-related skills-building and other supportive services that in some instances may help clients improve their HIV outcomes despite the ongoing presence of major or multiple barriers.[36]

PLWH who were in the consistently suppressed or inconsistently suppressed baseline groups have already by definition experienced some treatment success and were thus previously engaged in medical care and adhering to treatment to some degree. While the CCP may work better than usual care for PLWH with initial hurdles to ART access and adherence, the lack of a significant short-term CCP effect among the two groups with prior evidence of viral suppression suggests that the CCP is not more effective than usual care at helping people who have already established HIV care and treatment adherence to re-establish or maintain that adherence over a 12-month period. Among PWLH who were consistently or inconsistently suppressed throughout the prior year, the comprehensive and intensive approach of the CCP may not typically be needed (or wanted enough to be optimally utilized), and thus may not offer a real advantage over other forms of support available under NYC usual care.

Additionally, the NYC epidemic has shown substantial improvements in VLS over time [37], likely driven by advances in ART, treatment guideline expansion, and a robust local and state system of medical and non-medical services for PLWH. High-quality ‘usual care’ would tend to mute intervention effects in all baseline treatment status groups.

Case management interventions vary significantly in design and target population, making cross-study comparisons difficult.[19] Randomized trials of case management interventions have not evaluated VLS outcomes; however trial data suggest that case management (versus usual care) improves a) linkage to care among newly diagnosed PLWH and among PLWH who are leaving prison and b) retention in HIV medical care for PLWH with a history of inconsistent HIV care attendance [17, 20, 21]. To a degree our results align with and extend these findings; among PLWH who need *linkage* assistance and/or have been unable to achieve suppression at all during the past year, case management under this care coordination model is more effective than usual care at achieving VLS.

Cohort studies of case management interventions have evaluated VLS outcomes and reported null results.[15, 20] In a retrospective cohort evaluation, clients receiving care at clinics that provided Ryan White medical case management had significantly higher engagement in care but no greater VLS when compared to clients at clinics that did not provide Ryan White medical case management (adjusted Odds Ratio (aOR) 1.06; 95%Confidence Interval (CI): 0.68–1.62) [15]. In a prospective cohort study conducted among homeless and marginally housed PLWH, moderate case management (defined as case management in 25–75% of the study quarters, versus none or rare case management, defined as case management in <25% of study quarters) improved adherence to ART (adjusted β coefficient of 0.13 (95%CI, 0.02–0.25) but not viral suppression (aOR 1.4; 0.6–3.4))[18].Notably, however, these cohort studies did not present results stratified by baseline VLS status.

Strengths of our study include the use of a population-based data source to rigorously derive a contemporaneous observational comparison group, which was large enough to identify a non-CCP PLWH match (similar with regard to measured factors) for 96∙5% of the CCP sample. Additionally, deriving outcome information in an identical manner for CCP and non-CCP recipients from the longitudinal and population-based HIV Registry ensured that it was highly complete across the cohort and over time, regardless of care location or changes in care provider within NYC. Finally, our method’s explicit attention to matching on enrollment/pseudo-enrollment dates controlled for secular trends of increasing VLS in NYC over time.

Our study has several limitations. First, uncontrolled or poorly controlled confounding may exist, as this was an observational study. Since our propensity models were limited to variables available in the NYC HIV Surveillance Registry, we were unable to directly account for psychosocial factors known to be associated with poor VLS (e.g., homelessness, drug use, and mental illness), which may have been differential by arm even after propensity matching. Second, we were not able to describe or account for services or service delivery models received in the non-CCP comparison group, and we were not able to describe non-CCP services received in the CCP group. Third, we excluded CCP enrollees who had died in the first 12 months after enrollment, and exclusion of cohort members with late-stage disease and/or rapid disease progression may bias estimates.[38] The CCP aims to enroll persons with documented histories of or immediate risk for poor HIV outcomes. Specifically, persons with late-stage HIV disease may be enrolled in the CCP as a last attempt to stop rapid disease progression and avert mortality; some CCP agencies report recruiting for the CCP from their inpatient departments. However, there is no direct analog with non-CCP PLWH, since the pseudo-enrollment date assignment is random. To increase the comparability of CCP with non-CCP groups, we required all individuals in the cohort to have ≥12 months of observation beyond their pseudo-enrollment/enrollment. Fourth, not all program enrollment eligibility criteria could be translated to Registry-based eligibility criteria. For example, persons with comorbidities (e.g., depression) assessed as detrimental to adherence were eligible for enrollment in the CCP, but information on comorbidities is unavailable in the Registry. However, a high proportion (92%) of CCP persons specifically met the Registry-based eligibility criteria, which suggests we may have captured most of the non-CCP persons eligible for the CCP. Finally, our results may not be fully generalizable to Ryan White clients in settings outside NYC.

### Conclusions

New York’s Ryan White CCP intervention has shown a positive short-term effect on VLS among newly diagnosed PLWH and those who were consistently virally *un*suppressed in the year prior to the start of follow-up, suggesting the program may be effective at helping with initial hurdles to ART access and adherence. However, the absence of an effect among previously diagnosed persons with any suppression in the year prior to enrollment indicates the program confers no advantage over usual care for resuming or maintaining recent adherence. Efforts to refine CCP service delivery in NYC could better target for enrollment those newly diagnosed and those consistently *un*suppressed, and also enhance strategies for restoring and maintaining viral suppression (e.g., low-touch supports like texted dose reminders) among PLWH who have had prior treatment success.[39] Future studies should assess longer-term outcomes, including sustained viral suppression, in this population and others with known barriers to HIV care and treatment.

## Supporting information

S1 Table Definitions and examples of the HIV registry based eligibility criteria for the care coordination program. (DOCX)
