# Supplementary material for: Short-term effectiveness of HIV care coordination among persons with recent HIV diagnosis or history of poor HIV outcomes
Source: PLoS One. 2018 Sep 24;13(9):e0204017. doi: 10.1371/journal.pone.0204017 (PMC6152971; doi:10.1371/journal.pone.0204017)
Supplement: S1 Table — (DOCX) [file pone.0204017.s001.docx]

| SUPPLEMENTARY MATERIAL  Supplemental Table 1. Registry Based Eligibility Criteria: Definitions and Examples | | |
| --- | --- | --- |
| Registry Eligibility Definition | **Eligibility Window^a^** | **Example** |
| Newly diagnosed: Persons diagnosed from 12/01/2008 – 03/31/2013 | Eligibility begins the month of diagnosis and ends 12 months after diagnosis | Person X is diagnosed on 2/15/2010 and eligible from 2/2010 to 2/2011 |
| Not consistently in HIV medical care: Persons without any CD4 or VL laboratory tests reported for any 9 month period (after diagnosis) from 12/01/2007 to 03/31/2013 | Eligibility begins either 9 months after the first care event that indicated at least a 9 month gap had occurred or at the start of the enrollment window^d^, whichever comes later.  Eligibility ends two years after the first care event that indicates a 9 month gap occurred or at the end of the enrollment window, ^d^ whichever comes first  Persons may have multiple eligibility windows for being out of care. | Person X has a VL reported on 02/01/2011 and a second VL reported on 12/01/2011 (i.e., a > 9 month gap in care).  Person X is eligible from 11/2011 (9 months after the care event that indicated a gap) to 2/2013 (two years after the first care event that indicated a gap). |
| Treatment Naïve: Anyone who has ever had a CD4 count <200 as of 03/31/2013 but had not initiated ART^b^ as of the date of the CD4 <200. | Eligibility begins either on the date of the first CD4 count <200 or at the start of the enrollment window,^d^ whichever comes later.  Eligibility ends at the end of the enrollment window^d^ 03/31/2013) or at ART initiation whichever comes first. | Person X has a CD4 count <200 reported on 6/1/2010 and does not appear to have started ART^b^. On 02/28/2011, Person X appears to have initiated ART^b^.  Person X is eligible for CCP from 06/2010 to 02/2011. |
| Poor ART Adherence: Among persons who appeared to have initiated ART^b^ as of 3/31/2012, poor adherence was defined as not achieving viral suppression^c^ or not have any viral loads reported in the first 12 months after ART^b^ initiation (i.e., the *adherence assessment period*). We assessed for poor ART adherence continuously until 3/31/2013.  The 12 month *adherence assessment period* (distinct from eligibility window) started on 12/01/2008 or the day after the VL that indicated ART initiation, whichever came later. | Eligibility begins the day after the adherence assessment period (where we would have expected the person to achieve VL suppression) and ends at the earlier date of VL suppression or 3/31/2013.  If a person achieved VLS, we checked the adherence assessment period (12 month window that starts the day after the VL suppression date) for poor adherence. Eligibility would again start the day after the adherence assessment window and end at the earlier date of VL suppression of 3/31/2013.  Persons may have multiple eligibility windows for poor ART adherence. | Person Y initiates ART on 01/01/2010, and the second VL in the pair that indicates ART initiation occurs on 02/01/2010. We check for VL suppression^c^ (or an absence of viral loads) from 02/02/2010 to 02/01/2011 (i.e., the 12 month adherence assessment window).  Person Y achieved viral suppression on 8/15/2010. Person Y is eligible from 02/2010 to 08/2010. |
| VL Rebound:  Suppressed VL^c^ followed by 2 consecutive unsuppressed VL (>200 copies/mL) tests in 12 months following the suppressed VL  We looked for a rebound in viral loads reported from 12/01/2007 to 03/31/2013 | Eligibility either begins on the date of the second unsuppressed viral load or at the start of the enrollment window,^d^ whichever comes later.  Eligibility ends at evidence of durable VL suppression (2 consecutive suppressed VLs at least 14 days apart). If no durable VL suppression then eligibility ends at the earlier of 12 months or 3/31/2013.  Persons may have multiple eligibility windows for VL rebound. | Person Y has a suppressed VL reported on 8/15/2010, an unsuppressed VL reported on 12/10/2010, and an unsuppressed VL reported on 02/20/2011. Person Y had no evidence of durable suppression after 02/20/2011.  Person Y is eligible from 02/2011 to 02/2012 |
| High VL: Persons with a VL ≥ 10,000 copies/mL reported from 12/01/2008 – 03/31/2013 | Eligibility begins at the start of the enrollment window^d^ or when the high VL is reported, whichever comes later. Eligibility ends 12 months after the high VL is reported.  Persons may have multiple eligibility windows for high VL. | Person Y has a high VL reported 10/15/2011.  Person Y is eligible from 10/2011 to 10/2012 |
| ART antiretroviral treatment; VL viral load   1. For patients who died, eligibility windows end 12 months prior to the date of death. 2. ART initiation was defined as the first occurrence of EITHER a ≥1-log drop in viral load within 3 months of a previous viral load measurement, OR a suppressed VL (≤200 copies/mL). 3. VL ≤200 copies/mL 4. Enrollment window starts on December 1, 2009 and ends March 31, 2013. | | |
